# Contributing factors to patient safety incidents at three selected public hospitals in Gauteng province, South Africa: Perspectives from nursing staff and quality assurance managers

**DOI:** 10.4102/curationis.v49i1.2801

**Published:** 2026-01-23

**Authors:** Lowani R. Serongwa, Kholofelo L. Matlhaba

**Affiliations:** 1 Department of Health Studies, Faculty of Human Sciences, University of South Africa, Pretoria, South Africa

**Keywords:** contributing factors, nursing staff, patient safety, patient safety incident, public hospital, quality assurance managers

## Abstract

**Background:**

Patient safety incidents are a major concern in healthcare delivery, impacting patient care and health outcomes. Understanding the factors contributing to patient safety incidents is important for developing targeted strategies. Identifying these factors will enable healthcare organisations to formulate effective approaches to prevent and mitigate incidents.

**Objectives:**

This study aimed to assess the contributing factors to patient safety incidents at the three selected public hospitals in Gauteng province, South Africa. The research was conducted at the three selected public hospitals in Gauteng province, South Africa, categorised as central, regional and district.

**Method:**

A descriptive quantitative approach was utilised. Five hundred questionnaires were administered to nursing staff and quality assurance managers. A stratified sampling strategy was employed. The data were analysed using the Statistical Package for Social Sciences (SPSS) Version 26.0.

**Results:**

Work or environment (92.72%), organisational or service (91.00%), staff (87.40%) and patient (90.97%) factors were identified as the contributing factors to patient safety incidents.

**Conclusion:**

The overall impression of these results is that multiple factors contribute to patient safety incidents, with varying levels of perceived impact. The findings of the study imply the need for policy development, provision of adequate staffing, consumables and equipment, staff training on developed policies and procedures, and embarking on continuous quality improvement initiatives.

**Contribution:**

This study contributes to the understanding of factors contributing to patient safety incidents within the public hospitals and furnishes healthcare leaders with focused areas for improvement.

## Introduction

A patient safety incident is an unplanned or unintended event or circumstance that could have resulted or did result in harm to a patient while in the care of a health facility (National Guideline for Patient Safety Incident Reporting and Learning in the Health Sector of South Africa 2022). The World Health Organization (2020) indicated that globally, as many as 4 out of 10 patients are harmed in primary and ambulatory care settings while receiving healthcare.

From the study conducted in England and Wales by Urquhart et al. (2021), the identifiable contributory factors for the occurrence of patient safety incidents were: failure to follow protocols, staff mistakes, a lack of active decision-making and communication between teams. Inadequate working environment and lack of staff were identified as the contributing factors to the occurrence of patient safety incidents in a study conducted at a teaching hospital in the Žilina region in Slovakia (Kohanová et al. 2023).

Li et al. (2024) revealed that leadership behaviour and job burnout contributed to patient safety incidents from their study performed at a hospital in Shiyan City, Hubei province, China. An analysis of patient safety incidents in public hospitals in Hidalgo, Mexico, (Castro-del Ángel 2024) identified patient characteristics and the application of protocols as factors contributing to patient safety incidents. The study conducted in Chilean hospitals by Dueñas et al. (2023) found that ineffective interprofessional communication, non-adherence to protocols and lack of personnel contributed to patient safety incidents. Mujuru and Peisah (2024) identified communication lapses and an implementation gap between standards and practice as the factors contributing to patient safety incidents from their study conducted at the public hospitals in Australia.

Staff factors, patient-related factors, lack of or faulty equipment, communication issues and a lack of policies and/or guidelines were identified as the contributing factors to patient safety incidents in a study conducted at the Kenyatta National Hospital in Kenya (Okutoyi et al. 2024). For South Africa, the top four contributing factors to patient safety incidents during the financial year 2022/23 were patient factor (78%), followed by staff factors (33%), work factors (17%) and organisational or service factors (13%) (National annual patient safety incidents, complaints, compliments, and suggestions report 2023). From the study conducted at a hospital in KwaZulu-Natal province in South Africa, Mgobozi and Mahomed (2021) found that patient factors, staff factors, suboptimal adherence to protocols for patient safety and inadequate equipment were the contributing factors to patient safety incidents.

Patient safety incidents can have far-reaching effects, impacting patients, healthcare workers, and healthcare systems equally. These patient safety incidents can result in physical harm, emotional trauma, and financial burdens, eventually compromising the quality of life for patients. Healthcare workers, on the other hand, may suffer from emotional distress, professional repercussions, and increased workload, probably affecting their well-being and job satisfaction. Moreover, healthcare systems may suffer significant financial costs, reputational damage, operational disruptions, and regulatory scrutiny, underscoring the necessity of robust patient safety measures. The multifaceted impact of patient safety incidents emphasises the importance for healthcare organisations to prioritise patient safety and quality care.

This study focuses on the unique challenges and contributing factors specific to Gauteng province, allowing for targeted interventions. Recognition of the contributing factors to patient safety incidents is essential for formulating focused strategies to prevent and mitigate the occurrence of these incidents. Through investigation of the causes of patient safety incidents, this research seeks to contribute to the development of safer healthcare settings and improved patient outcomes.

### Purpose of the study

The purpose of the study was to determine the contributing factors to patient safety incidents at the three selected public hospitals in Gauteng province, South Africa.

### Research question

The following research question was addressed in this study:

- What are the contributing factors to patient safety incidents at the three selected public hospitals in Gauteng province, South Africa?

### Objectives of the study

The objectives of the study were as follows:

- to identify the work or environment factors contributing to patient safety incidents at the three selected public hospitals in Gauteng province, South Africa
- to examine the organisational or service factors contributing to patient safety incidents at the three selected public hospitals in Gauteng province, South Africa
- to explore the staff related factors contributing to patient safety incidents at the three selected public hospitals in Gauteng province, South Africa
- to review the patient-related factors contributing to patient safety incidents at the three selected public hospitals in Gauteng province, South Africa.

## Research methods and design

### Study design

Quantitative descriptive design was used to assess the contributing factors to patient safety incidents.

### Study setting

The research was carried out at the three selected public hospitals in Gauteng province, South Africa, categorised as one central hospital, one regional hospital and one district hospital. By including hospitals from different levels (central, regional, district), the study captures a spectrum of healthcare delivery settings, reflecting varying capacities, patient volumes, and service complexities. Each hospital type serves different patient demographics and case mixes, allowing for broader insights into patient safety incidents across diverse contexts. Central, regional, and district hospitals often differ in resource allocation, staffing, equipment, and infrastructure, enabling the study to explore how these factors influence patient safety. Including hospitals at different functioning levels facilitates comparisons of patient safety incident contributing factors, potentially highlighting setting-specific challenges.

### Study population

According to LoBiondo-Wood and Haber (2022), a population is a well-defined population set possessing specified properties or characteristics from which data can be collected and analysed. The study population consisted of professional nurses, enrolled nurses, enrolled nursing assistants, operational managers, assistant managers nursing, and quality assurance managers who were available, willing, and consenting to participate in the study while meeting the established inclusion criteria. Nursing staff are directly involved in patient care, positioning them to observe and experience factors contributing to patient safety incidents firsthand. Nurses play a pivotal role in ensuring patient safety, making their perspectives invaluable for understanding factors contributing to patient safety incidents. Quality assurance managers are responsible for overseeing and improving quality and safety processes, granting them insight into systemic factors impacting patient safety. Including both nursing staff and quality assurance managers captures a range of viewpoints, enriching the study’s findings on the factors contributing to patient safety incidents. Eligible participants included operational managers nursing, assistant managers nursing, quality assurance managers, professional nurses, enrolled nurses, and enrolled nursing assistants who met the following criteria: had a tenure of 1 year or more at the study site, were willing and provided consent to participate in the study and were on duty during the data collection period.

### Sample size

Sample size is the total number of participants or units in the sample (Khan et al. 2023). The Raosoft online sample size formula was utilised to ascertain the required sample size. A minimum recommended sample size of 356 respondents was necessary, based on an anticipated 50% response rate, a 95% confidence level, and a projected margin of error of 5%.

Table 1 indicates the estimated participants as follows: professional nurses (110), enrolled nursing assistants (88), enrolled nurses (83), operational managers nursing (42), assistant managers nursing (25) and quality assurance managers (8). The reason to have this number of participants from the central hospital was to ensure representation of diverse units and staff roles within the central hospital. The rationale for the number of participants at both the regional hospital and district hospital was to ensure better representation.

**TABLE 1:** Number of recommended participants from the three public hospitals.

Position	Central hospital	Regional hospital	District hospital	Total
Assistant Manager Nursing	15	5	5	25
Operational Manager Nursing	20	12	10	42
Quality Assurance Manager	6	1	1	8
Professional Nurse	40	40	30	110
Enrolled Nurse	29	30	24	83
Enrolled Nursing Assistant	30	32	26	88

**Total**	**140**	**120**	**96**	**356**

*Source:* Adapted from Famolaro, T., Yount, N.D., Burns, W., Flashner, E., Liu, H. & Sorra, J., 2016, *Hospital survey on patient safety culture: 2016 user comparative database report, AHRQ Publication No. 16-0021-EF*, Agency for Healthcare Research and Quality, Rockville, MD.

A total of 436 respondents, including professional nurses, enrolled nurses, enrolled nursing assistants, operational managers, assistant managers nursing, and quality assurance managers, completed the questionnaires.

### Sampling strategy

Sampling strategy entails selecting a representative subset of the target population to collect data (Khan et al. 2023). A stratified sampling strategy was employed as the study involved participants from three different hospital settings (strata) in Gauteng province, South Africa. Non-proportional stratified sampling was used as the sample sizes from the strata were not proportional to their population sizes. The sampling fractions differed as follows: central hospital (230 sampled from 419 total population = 55%), regional hospital (150 sampled from 172 total population = 87%), and district hospital (120 sampled from 148 total population = 81%). Simple random sampling was used within the strata to get the required participants.

### Data collection

McKenna and Copnell (2024) define data collection as the process of gathering data to respond to a research question. According to Khan et al. (2023), a questionnaire can be described as a structured compilation of questions administered to a sample of the population from which required information is sought. The questionnaire was structured into five sections. Section A covered demographic characteristics, while Section B concentrated on staff factors contributing to patient safety incidents. Section C focused on patient factors contributing to patient safety incidents and Section D addressed work or environment factors contributing to patient safety incidents. Finally, Section E dealt with organisational or service factors contributing to patient safety incidents. Collectively, these sections encompassed the contributing factors to patient safety incidents as perceived by the nursing staff and quality assurance managers at the three selected public hospitals.

A research assistant who holds a Master of Nursing qualification provided a link of digital questionnaires and hard copies of the questionnaires to the target population. The data collection process was commenced in July 2024 and completed in November 2024.

### Validity and reliability of data collection tool

A comprehensive literature review preceded the development of the instrument, following which the instrument underwent pre-testing at a public hospital excluded from the current study. The clearly constructed questionnaire underwent verification by the supervisor and was subsequently pretested at a public hospital not included in this study.

### Data analysis

Data analysis pertains to the process of analysing gathered data to draw conclusions (McKenna & Copnell 2024). With the help of the statistician, data were analysed using the Statistical Package for Social Sciences (SPSS) Version 26.0. As observed by Aljohani et al. (2021), descriptive statistics represent a mathematical method utilised to synthesise, summarise, and describe data gathered from a sample through frequency and percentage distributions. These statistics were employed to summarise the overall distribution and identify contributing factors to patient safety incidents at the three selected public hospitals located in Gauteng province, South Africa.

Pearson’s chi-square test was utilised to assess the association between two categorical variables. The findings were evaluated at a 0.05 significance level, indicating that the results were considered significant if the observed *p*-value fell below 0.05.

### Ethical considerations

Ethical principles are the moral guidelines that researchers must adhere to for protecting the rights and welfare of human participants. The four fundamental principles of research include the principles of beneficence, nonmaleficence, justice and autonomy (Sukhpal & Muthuvenkatachalam 2024).

Researchers have an ethical duty to prioritise the well-being and interests of research respondents. To comply with the principle of beneficence, researchers must ensure that the potential benefits of the research clearly surpass any potential risks or harms (Sukhpal & Muthuvenkatachalam 2024). Ethical clearance to conduct this study was obtained from the University of South Africa College of Human Science Ethics Committee (reference number: 41324722_CREC_CHS_2024). No respondent was exposed to any harmful situation. Data collection took place in designated hospital boardrooms, providing a secure and private setting. Participants also had the flexibility to complete the questionnaire at a time and location of their convenience.

Researchers have an ethical duty to make every effort to avoid causing harm to research respondents. To comply with the principle of nonmaleficence, researchers must take proactive steps to reduce the risks associated with the research (Sukhpal & Muthuvenkatachalam 2024). Respondents were not exposed to any harm. Data collection took place in designated hospital boardrooms, providing a secure and private setting.

Researchers have an ethical obligation to ensure that respondents are treated fairly throughout all facets of the research process. This means that all individuals who meet the study’s inclusion criteria should be afforded an equal opportunity to participate without favouritism or discrimination (Sukhpal & Muthuvenkatachalam 2024). Inclusion criteria were used to select the respondents. Eligible participants included operational managers nursing, assistant managers nursing, quality assurance managers, professional nurses, enrolled nurses, and enrolled nursing assistants who met the following criteria: had a tenure of 1 year or more at the study site, were willing and provided consent to participate in the study and were on duty during the data collection period.

As stated by Sukhpal and Muthuvenkatachalam (2024), researchers must provide respondents with comprehensive information about the study, encompassing the risks and benefits, before getting their consent. Furthermore, respondents have the right to make their own decisions about their participation in research. Lastly, respecting human dignity means treating respondents with respect, fairness, and recognising their intrinsic value. The respondents signed the consent forms freely and willingly.

The researcher secured ethical approval and permission to conduct the study from the Research Ethics Committee of the public university located at the northern part of Gauteng province. In addition, consent to proceed with the study was granted by the management of the three selected public hospitals located in Gauteng province, South Africa.

## Results

The results were presented under the headings: the number of questionnaires sent out and response rate from each hospital, demographic characteristics of the study population and contributing factors to patient safety incidents.

Table 2 indicates that most of the respondents were from the central hospital, with 206 respondents (90%) out of 230 questionnaires sent out. The regional hospital had a response rate of 85.33% and lastly, the district hospital had a response rate of 85%.

**TABLE 2:** The number of questionnaires sent out and the response rate from each hospital.

Hospital category	Number of the questionnaires sent out	Number of the questionnaires returned	Response rate (%)
Central hospital	230	206	90.00
Regional hospital	150	128	85.33
District hospital	120	102	85.00

**Total**	**500**	**436**	**87.00**

*Source:* Adapted from South Africa. Department of Health, 2012, *Regulations relating to categories of hospitals*, Government Printers, Pretoria.

According to Table 3, the respondent profile indicates professional nurses formed the largest group, comprising 181 of 436 participants (41.5%). Enrolled nurses followed with 96 respondents (22.0%), and enrolled nursing assistants accounted for 72 respondents (16.5%). Operational managers nursing numbered 50 respondents (11.5%), and assistant managers nursing represented 28 participants (6.4%). Quality assurance managers constituted the smallest cohort, with 9 respondents (2.1) participating in the study.

**TABLE 3:** The demographic characteristics of the study population.

Position	Central hospital		Regional hospital		District hospital	
	*n*	%	*n*	%	*n*	%
Assistant Manager Nursing	16	7.77	7	5.47	5	4.90
Operational Manager Nursing	32	15.53	8	6.25	10	9.80
Quality Assurance Manager	6	2.91	2	1.56	1	0.98
Professional Nurse	108	52.43	43	33.59	30	29.41
Enrolled Nurse	41	19.90	30	23.44	25	24.51
Enrolled Nursing Assistant	3	1.46	38	29.69	31	30.40

**Total**	**206**	**100**	**128**	**100**	**102**	**100**

*Source:* Adapted from Famolaro, T., Yount, N.D., Burns, W., Flashner, E., Liu, H. & Sorra, J., 2016, *Hospital survey on patient safety culture: 2016 user comparative database report, AHRQ Publication No. 16-0021-EF*, Agency for Healthcare Research and Quality, Rockville, MD.

Table 4 presents the contributing factors to patient safety incidents with work or environment factors leading at 92.72% (consumables [not available or insufficient] 94.51%, equipment [not available or not functioning] 93.30%, physical environment/infrastructure [damaged or worn or inadequate or inappropriate] 93.98% and safety or security [security systems insufficient] 89.08%), followed by organisational or service factors at 91 (staffing [staffing shortages] 96.02%, bed utilisation [unavailability of beds] 93.30%, clinical protocols or policies or procedures [not available or up to date or not approved] 89.70% and package of services [package of services not offered or cancellation or unavailability of services] 84.97%).

**TABLE 4:** Contributing factors to patient safety incidents for three public hospitals.

Main classification	Sub-classification	Strongly agreed and agreed responses (%)			Average percentage of strongly agreed and agreed responses	*p*-value
		Central hospital	Regional hospital	District hospital		
Work or environment factors	Consumables (not available or insufficient)	97.39	95.45	90.70	94.51	0.029
	Equipment (not available or not functioning)	96.73	93.64	89.53	93.30	0.000
	Physical environment or infrastructure (damaged or worn or inadequate or inappropriate)	94.77	91.82	95.35	93.98	0.045
	Safety or security (security systems insufficient)	94.77	94.55	77.91	89.08	0.000
	Average percentage of strongly agreed and agreed responses	95.92	93.87	88.37	92.72	0.019
Organisational or service factors	Staffing (staffing shortages)	98.69	96.36	93.02	96.02	0.001
	Bed utilisation (unavailability of beds)	96.08	95.45	88.37	93.30	0.018
	Clinical protocols or policies or procedures (not available or up to date or not approved)	93.46	87.27	88.37	89.70	0.018
	Package of services (package of services not offered or cancellation or unavailability of services)	92.81	90.00	72.09	84.97	0.000
	Average percentage of strongly agreed and agreed responses	95.26	92.27	85.46	91.00	0.009
Staff factors	Social factors (stress, a lack of motivation, high workload, fatigue)	96.08	93.64	95.35	95.02	0.000
	Human error – clinical (technical errors made while performing the procedure or not performing the clinical procedure as required)	94.12	91.82	86.05	90.66	0.000
	Human error – administrative (technical error made while performing administrative procedure or not performing the administrative procedure as required)	94.77	90.00	73.26	86.01	0.000
	Risky or reckless behaviour (risky, reckless because of forgetfulness, fatigue, overconfidence) or criminal act	90.85	87.27	89.53	89.22	0.000
	Communication factors (language difficulties, poor communication, health literacy)	91.50	81.82	81.40	84.91	0.000
	Leadership (a lack of supervision, delegation of duties outside of scope of practice)	92.16	90	53.49	78.55	0.000
	Average percentage of strongly agreed and agreed responses	93.25	89.09	79.85	87.40	0.000
Patient factors	Condition/disease-related factor (problems with substance abuse other mental illness, spasticity, cognitive fall outs post Cerebral Vascular Accident [CVA], existing comorbidities)	91.50	93.02	88.18	90.90	0.003
	Behaviour (risky, reckless, overconfident, criminal act, attention issues [absentmindedness or forgetfulness, distraction], fatigue or exhaustion)	92.16	89.09	91.86	91.04	0.253
	Average percentage of strongly agreed and agreed responses	91.83	91.06	90.02	90.97	0.128

*Source:* Adapted from South Africa. Department of Health, 2022, *National guideline for patient safety incident reporting and learning in the health sector of South Africa*, Government Printers, Pretoria.

Patient factors were rated 90.97% (condition or disease-related factor [problems with substance abuse other mental illness, spasticity, cognitive fall outs post Cerebral Vascular Accident (CVA), existing comorbidities] 90.90% and behaviour [risky, reckless, overconfident, criminal act, attention issues (absentmindedness or forgetfulness, distraction, fatigue or exhaustion] 91.04%).

Finally, staff factors were scored 87.40% (social factors [stress, a lack of motivation, high workload, fatigue] 95.02%, human error – clinical [technical errors made while performing the procedure or not performing the clinical procedure as required] 90.66%, human error – administrative [technical error made while performing administrative procedure or not performing the administrative procedure as required] 86.01%, risky or reckless behaviour [risky, reckless due to forgetfulness, fatigue, overconfidence or criminal act] 89.22%, communication factors [language difficulties, poor communication, health literacy] 84.91% and leadership [lack of supervision, delegation of duties outside of scope of practice] 78.55%).

Work or environment factors (0.019), organisational or service factors (0.009) and staff factors (0.000) revealed statistically significant differences across the three public hospitals. Differences in hospital infrastructure, equipment, workload, staffing levels, leadership styles, communication and guidelines might be responsible for these variations in these three public hospitals.

Patient factors (0.128) did not differ significantly across the three public hospitals. These findings might be because of the similar types of health conditions or diseases prevalent among the patients across the three public hospitals.

## Discussion

Consumables, equipment, the physical environment, and security or safety are work or environment factors that contribute to patient safety incidents. According to Patel, Cieslak and Hertig (2023), two major healthcare supply chain issues that contribute to patient safety incidents are product delays and drug shortages. Product delays may be medical products such as active and inactive pharmaceutical ingredients, as well as syringes, gloves, masks, needles, testing materials, and other personal protective equipment. Mohammadi, Rustaee and Bijani (2023), identified lack of standard equipment and adequate physical space as the contributing factors to patient safety incidents. The physical environment (23.3%) was identified as the contributing factor to patient safety incidents (South Africa 2025:1). Unguarded doors or windows permit the patient to flee from the unit in inpatient psychiatry (Donaldson et al. 2021). Limited budgets, ageing healthcare facilities, delays in acquiring necessary supplies and equipment, and an inadequate number of security officers might be work or environment factors contributing to the patient safety incidents.

The main organisational or service factors contributing to patient safety incidents were staffing, bed utilisation, clinical protocols or policies or procedures and package of services. A shortage of human resources has been identified as a contributing factor to patient safety incidents (Halinen et al 2024). Kwobah et al. (2023), discovered that insufficient bed capacity contributes to the occurrence of patient safety incidents. Lack of clinical guidelines or protocols on patient safety management was discovered as the contributing factor to patient safety incidents (Mohammadi et al. 2023). Reported or cancelled care because of unavailable or closed services contributes to the occurrence of patient safety incidents (Fournier et al. 2021). Staff shortages, insufficient matching of staff skills to patient needs, high bed occupancy rates, outdated clinical guidelines, non-adherence to the established protocols and procedures, and the absence of needed services might be the organisational or service factors contributing to patient safety incidents.

Social factors, human error – clinical, human error – administrative, risky or reckless behaviour, communication factors and leadership were rated highly as the most staff factors contributing to the occurrence of patient safety incidents. Gauteng Department of Health report on patient safety incident contributory factors (South Africa 2025:1) showed that human error – clinical (39.2%) and human error – administrative (7.6%) contributed to patient safety incidents. Adu and Zuma (2024) revealed that the majority of the study participants linked patient safety incidents to poor communication pathways within the organisational structure and among multidisciplinary staff. Adriansyah et al. (2022), on analysis of the quality of supervision on patient safety incidents, found that poor quality of supervision led to a high incidence of patient safety incidents. This finding is corroborated by Amaniyan et al. (2020) who discovered that a lack of supervision of the work process led to patient safety incidents. Collaboration challenges, insufficient training or experience in complex clinical tasks, non-adherence to established clinical guidelines, weaknesses in administrative systems (for example, poor documentation), complacency or disregard for patient safety protocols, poor communication among healthcare teams, and insufficient strategic direction on patient safety might be the reasons for the above findings.

Condition/disease-related factor and behaviour were identified as the primary patient factors contributing to patient safety incidents. Alcohol and substance abuse and unstable mental state were identified as the contributing factors of patient safety incidents (Kwobah et al. 2023). Gauteng Department of Health report on patient safety incident contributory factors (South Africa 2025:1) showed that patient behaviour (33.6%) contributed to patient safety incidents. Patients with complex, multiple, or severe diseases might be more prone to safety incidents because of the complexity in management. The presence of multiple health issues complicates care and increases incident risk. Patients not following prescribed regimens (e.g., strict bed rest) might experience a patient safety incident.

### Strengths and limitations

The descriptive quantitative method employed in the study offers notable advantages, including the reduction of personal bias through the collection of numerical data; moreover, it yields consistent results when replicated in various settings, thereby enhancing the reliability of the findings. However, it is crucial to acknowledge the study’s limitations, as it was conducted at only three selected public hospitals in Gauteng province, South Africa, consequently restricting the generalisability of the results to all public hospitals in the province or the entire country. Furthermore, the scope of the results is also confined to the perspectives of the nurses and quality assurance managers who participated in the study, indicating that broader applicability would require additional research encompassing a wider range of institutions and stakeholders.

### Recommendations

To enhance patient safety in public hospitals, several strategic interventions are essential. Ensuring appropriate distribution of resources for consumables, equipment, infrastructure maintenance, and dedicated patient safety initiatives is critical. Establishing and maintaining optimal staffing ratios can help alleviate staff shortages and mitigate excessive workloads, supporting better care delivery. Formulating and regularly reviewing evidence-based clinical protocols and procedures ensures they remain current and effective. Maintaining infrastructure addresses safety hazards posed by damaged or worn-out facilities, contributing to a safer environment. Regular staff training focusing on patient safety and adherence to clinical protocols is vital for competency and compliance. Sufficient supervision coupled with accountability measures can curb reckless behaviour, promoting a culture of responsibility. In addition, further research is warranted to comprehensively assess the primary contributing factors to patient safety incidents across all public hospitals in Gauteng province, South Africa, informing targeted interventions.

## Conclusion

This study identified various factors contributing to patient safety incidents, including systemic issues such as unavailability of consumables, equipment, and infrastructure, as well as human factors such as stress, fatigue, and human error. The findings also highlighted the importance of effective leadership, communication, and clinical protocols in preventing and mitigating the occurrence of patient safety incidents.

The multiplicity of these factors highlights the need for a multifaceted, focused strategic approach to enhance patient safety. Healthcare organisations must address these contributing factors through a combination of policy development, staff training, and embarking on continuous quality improvement initiatives. By understanding and addressing the root causes of patient safety incidents, healthcare providers can minimise the risk of patient safety incidents and improve patient outcomes.
